# Validation of Questionnaire Methods to Quantify Recreational Water Ingestion

**DOI:** 10.3390/ijerph15112435

**Published:** 2018-11-01

**Authors:** Laura M. Suppes, Kacey C. Ernst, Leif Abrell, Kelly A. Reynolds

**Affiliations:** 1 Environmental Public Health Program, The University of Wisconsin-Eau Claire, 105 Garfield Avenue, Eau Claire, WI 54702, USA; 2 Mel and Enid Zuckerman College of Public Health, The University of Arizona, P.O. Box 245163, Tucson, AZ 85724, USA; Kernst@email.arizona.edu (K.C.E.); Reynolds@email.arizona.edu (K.A.R.); 3 Department of Soil, Water & Environmental Science, The University of Arizona, Gould-Simpson Building Room 611, 1040 East 4th Street, Tucson, AZ 85721, USA; Abrell@email.arizona.edu

**Keywords:** pool water ingestion, recreational water, swimming pool, risk assessment

## Abstract

Swimming pool water ingestion volumes are necessary for assessing infection risk from swimming. Pool water ingestion volumes can be estimated by questionnaire or measuring a chemical tracer in swimmer urine. Questionnaires are often preferred to the chemical tracer method because surveys are less time consuming, but no research exists validating questionnaires accurately quantify pool water ingestion volumes. The objective of this study was to explore if questionnaires are a reliable tool for collecting pool water ingestion volumes. A questionnaire was issued at four pool sites in Tucson, Arizona to 46 swimmers who also submitted a urine sample for analyzing cyanuric acid, a chemical tracer. Perceived ingestion volumes reported on the questionnaire were compared with pool water ingestion volumes, quantified by analyzing cyanuric acid in swimmer urine. Swimmers were asked if they swallowed (1) no water or only a few drops, (2) one to two mouthfuls, (3) three to five mouthfuls, or (4) six to eight mouthfuls. One mouthful is the equivalent of 27 mL of water. The majority (81%) of swimmers ingested <27 mL of pool water but reported ingesting >27 mL (“one mouthful”) on the questionnaire. More than half (52%) of swimmers overestimated their ingestion volume. These findings suggest swimmers are over-estimating pool water ingestion because they perceive one mouthful is <27 mL. The questionnaire did not reliably collect pool water ingestion volumes and should be improved for future exposure assessment studies. Images of the ingestion volume categories should be included on the questionnaire to help swimmers visualize the response options.

## 1. Introduction

The annual number of Recreational Water Illness (RWI) outbreaks associated with treated recreational water venues (“pools”) in the U.S. has increased since 1978 when reporting was initiated (pools are defined as swimming pools, spas, interactive fountains, wading pools and dive pools) [1,2,3]. RWIs range from acute gastrointestinal illness (AGI), skin infection or rash to acute respiratory illness (ARI). The majority of outbreaks are associated with AGI, which accounted for 81% of outbreaks during summer months in 2011–2012 [4]. Most AGI outbreaks in treated recreational water are associated with ingesting *Cryptosporidium*. *Cryptosporidium* has been detected in treated recreational water and associated with outbreaks internationally [5,6,7,8]. From 2000–2014, *Cryptosporidium* caused 58% of treated recreational water outbreaks in the U.S. [9]. The volume of pool water ingested by swimmers is necessary to quantify infection risk from enteric pathogens like *Cryptosporidium* [10]. Risk assessment can help identify unsafe swimming behaviors, at-risk populations, and priority hazards to direct the development of pool safety guidelines. Recognizing the need for accurate data collection tools for swimming pool risk assessment, this study compared perceived ingestion volumes reported on a questionnaire to pool water ingestion volumes quantified by analyzing cyanuric acid in swimmer urine. The questionnaire merged information and survey questions collected and developed by the Centers for Disease Control and Prevention (CDC), the U.S. Environmental Protection Agency (USEPA), and academic researchers to assess a variety of swimmer exposures. The objective was to determine if questionnaires are a reliable tool for collecting pool water ingestion volumes.

One primary exposure related to risk of RWI is ingestion of water. Previously, the World Health Organization (WHO) used questionnaires to estimate swimming ingestion rates and found swimmers reported swallowing 20–50 mL/h [11]. These self-reported values, however, are underestimated when compared to ingestion ranges found in other studies applying quantitative measurement techniques. Thus, the WHO questionnaire may not accurately capture pool water ingestion magnitudes among swimmers.

Ingestion can be quantified using methods that compare cyanuric acid in urine and pool water. Cyanuric acid is added as a chlorine stabilizer to outdoor pool water, and when ingested, passes through the human body unmetabolized [12]. Controlled studies show 98% of cyanuric acid ingested is excreted in a 24 h period [12]. Using this technique, researchers Dufour et al. and Suppes et al. showed swimmers ingested between 0–154 mL/h and 0–105.5 mL/h, respectively [13,14]. Information on perceived ingestion by study participants was not collected in the Dufour study, but was collected by Suppes et al. using the questionnaire discussed in this article (see Supplementary Materials). The questionnaire asked swimmers how much pool water was ingested during a timed swim. The current article is one part of the Suppes et al. study and describes how accurately swimmers perceive pool water ingestion by comparing reported to measured volumes. Our findings demonstrate swimmers perceive higher ingestion exposures than in reality, which explains why self-reported ingestion estimates are different than measured estimates.

## 2. Materials and Methods

### 2.1. Questionnaire Development

The CDC and USEPA websites and peer-reviewed literature were searched for pool outbreak survey tools, tools developed in response to outbreaks, and tools designed to capture swimmer exposures [15,16]. The CDC National Outbreak Reporting System (NORS) is available for reporting nationwide waterborne disease outbreaks and includes exposure questions related to recreational water. In-depth survey tools are also available through the CDC that collect data on swimmer activity, gastrointestinal symptoms, confounding exposures, pool operations and maintenance, and are designed to be administered by outbreak investigators [15]. Surveys intended to collect additional exposure information, such as potential disinfection by-product exposures, were reviewed from the USEPA assessment tool SWIMODEL among others [15].

Exposure risk factors relative to swimmer behavior and pool maintenance from the CDC surveys, SWIMODEL, and peer-reviewed literature were compiled and organized into a draft questionnaire. Three panels were assembled to review the draft for comprehensiveness and to recommend formatting and included (1) six experts from the swimming pool industry; (2) an international group of nine microbiologists, exposure scientists, and epidemiologists; and (3) an internal University of Arizona panel of six respiratory health, epidemiology, exposure science, and public health specialists. Meetings with each panel were held once and lasted 1–2 h following advance reviews of the questionnaire. Individual communication with panel members by email or phone occurred throughout the questionnaire development process. Questions from the draft were entered into DatStat Illume Survey Developer Gateway Version 5.1.1.17347 (Seattle, WA, USA). The questionnaire was further evaluated by the external review panel for errors and comprehensiveness prior to use. A modified version of the questionnaire can be viewed in Table S1 of the Supplementary Materials.

The question used in this study to estimate pool water ingestion by “mouthfuls/swim” was developed by Schets et al. and was selected over other surveys based on recommendations from the expert questionnaire review committees [17]. Other surveys used specific volume classifications, like “teaspoon”, that may have been difficult for younger participants in this study to interpret. The Schets study quantified the average volume in one mouthful (27 mL), which allowed measured volumes in the present study to be categorized into “mouthfuls/swim”. Swimmers were asked on our questionnaire if they swallowed (1) no water or only a few drops, (2) one to two mouthfuls, (3) three to five mouthfuls, or (4) six to eight mouthfuls. Using data from the Schets study indicating an average mouthful is 27 mL, qualitative variables from our questionnaire were converted to quantitative volumes. Despite the Schets study defining “no water to a few drops” as 0–5 mL, swimmers with measured ingestion between 0–26 mL were categorized as: “1: no water or only a few drops”. There was no qualitative ingestion category in the Schets study representing 6–26 mL. The other categories were: 27–54 mL (one to two mouthfuls), 55–135 mL (three to five mouthfuls), and 136–216 mL (six to eight mouthfuls).

### 2.2. Data Collection

This research was approved by the University of Arizona Human Subjects Research and Institutional Review Board (project number: 12-0272-12). The questionnaire was issued to 46 swimmers June–September 2013 in Tucson, Arizona, recruited at two outdoor public pools and two outdoor private pools. Swimmers arriving at the pools on data collection days were approached by a member of the research team, given details of the study’s objectives, and asked if they would participate by completing a questionnaire after swimming and submitting a 24 h urine sample to quantify pool water ingestion. Urine samples were preserved then cleaned by solid phase extraction and analyzed using ultra-high-pressure liquid chromatography tandem mass spectrometry (UHPLC-MS/MS) for cyanuric acid. Pool water samples were collected at each pool site on the day swimmers were recruited, transferred on ice, and preserved along with urine samples. Cyanuric acid was quantified in pool water using UHPLC-MS/MS. Pool water ingestion volumes were calculated using cyanuric acid concentrations in urine and pool water [13] (Equation (1)). Detailed results from the 24 h urine sample portion of this study are published elsewhere [14].

(1) $$\mathrm{water}\;\mathrm{ingestion}\;\left(L\right)\;=\;\left({\left[\mathrm{cyanuric}\;\mathrm{acid}\right]}_{\mathrm{urine}}\left(\frac{µg}{L}\right)÷{\left[\mathrm{cyanuric}\;\mathrm{acid}\right]}_{\mathrm{pool}\;\mathrm{water}}\left(\frac{µg}{L}\right)\right)\;\times \;\mathrm{urine}\;\mathrm{volume}\;\left(L\right)\;$$

All swimmers, regardless of age, gender, or other factors, were approached and asked to participate. Swim duration for all participants was recorded on the questionnaire. Participants accessed the questionnaire either on-site using tablets, electronic or smart phones, or on a personal computer through email. Questionnaires were completed within six hours of swimming.

## 3. Results

Thirty-eight of 46 participants had usable water ingestion values for analysis. Four did not submit a questionnaire, one submitted a urine sample less than the accepted volume threshold, and three urine samples had signal-to-noise ratios <3, which indicates a measurement below the analytical equipment limit of detection (UHPLC-MS/MS). The percent recoveries of cyanuric acid from urine and pool water were 6% and 112%, respectively. Table 1 summarizes the study population.

Table 2 illustrates the number of swimmers who correctly and incorrectly reported the volume range of pool water ingested during swimming. Sixteen of 38 swimmers (42%) correctly reported their ingestion volume, 20/38 (52%) overestimated the amount of pool water ingested and 2/38 (5%) underestimated their ingestion volume. Thirty-one of 38 swimmers (81%) actually ingested 0–26 mL of water, but only 11/38 swimmers (29%) correctly reported ingesting 0–26 mL. All swimmers (11/11) who reported ingesting “no water to a few drops” did ingest water within the volume range categorized as “no water to a few drops” (0–26 mL). Four of 20 swimmers who reported ingesting “one to two mouthfuls” actually ingested pool water within the volume range “one to two mouthfuls” (27–54 mL). Only one swimmer reported ingesting “three to five mouthfuls”, but six actually did ingest pool water within this volume range (55–135 mL). No swimmers ingested or reported ingesting 136–216 mL.

## 4. Discussion

**Figure 1:**
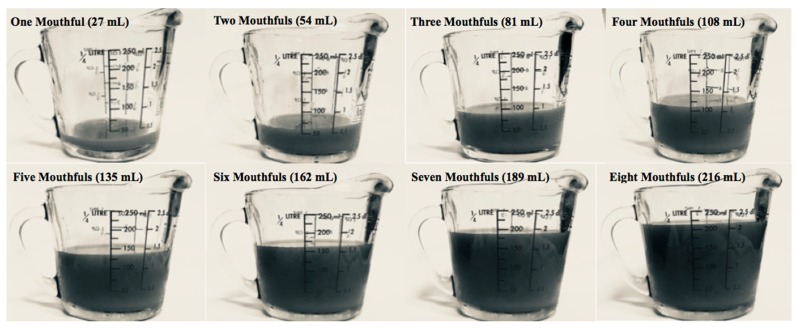
The figure illustrates one to eight mouthfuls of liquid in a one-cup/250 mL measuring glass, assuming one mouthful is equal to 27 mL of liquid [17].

Developers of the question used on our survey found the average volume of one mouthful to be 27 mL, which was used in this study to categorize measured ingestion volumes to mouthfuls. The majority (81%) of swimmers actually ingested <27 mL of pool water but reported ingesting >27 mL (one mouthful) on the questionnaire. More than half (52%) of swimmers overestimated their ingestion volume across all volume categories. These findings suggest swimmers are overestimating pool water ingestion because they perceive one mouthful to be <27 mL. The lack of accurate reporting of ingestion volumes using a question recommended by experts suggests a need for improving questionnaire techniques to assess recreational water ingestion. Since there is uncertainty about the volume of water in one mouthful, the questionnaire can be improved by including images of a one-cup/250 mL measuring glass with one to eight mouthfuls of liquid (Figure 1). Eight was the maximum number of mouthfuls on the questionnaire. The questionnaire can also be improved by changing the “no water to a few drops” category to “less than one mouthful” for consistency in questionnaire response options. Including Figure 1 would help swimmers visualize the ingestion volume categories to reduce inaccurate reporting.

Inconsistencies in method performance between this study and similar studies [13,18] and low recoveries of cyanuric acid in urine indicate a need for improving techniques to quantify pool water ingestion. Using comparable methods, Dorevitch et al. recovered 32.7% of cyanuric acid from swimmer urine and 96.5–99% of cyanuric acid from pool water [18]. Dufour et al. did not report recovery efficiencies for cyanuric acid in urine or pool water using a similar method [13]. Recovery of cyanuric acid in urine and pool water was 6% and 112%, respectively, in the current study. Like this study, Dorevitch et al. calculated pool water ingestion using Equation (1) and did not adjust cyanuric acid in pool water to account for the lower recovery in urine. Self-reported pool water ingestion quantities from a questionnaire by Dorevitch et al. were also compared to measured pool water ingestion quantities. To be consistent with Dorevitch and Dufour, no percent recovery adjustments were made to cyanuric acid in urine or pool water before analyzing measured and self-reported pool water ingestion in this study. Measured ingestion estimates could be higher than reported in all three studies, but exact pool water ingestion quantities cannot be estimated without a method that consistently recovers 100% of cyanuric acid in urine. Cyanuric acid extraction efficiencies are dependent on the solid phase extraction technique and analytical instrument. A more detailed comparison and discussion of method performance and limitations between this study and others is published elsewhere [14].

## 5. Conclusions

This study highlights the need for improved questionnaire techniques to assess recreational water ingestion. Our findings demonstrate swimmers perceive higher ingestion exposures than in reality, which explains why self-reported ingestion estimates are different than measured estimates from previous studies. Since there is uncertainty about the volume of water in one mouthful, researchers who use this question technique in the future should include images of a one-cup/250 mL measuring glass with one to eight mouthfuls of liquid to help swimmers visualize the ingestion volume categories. The questionnaire category “no water to a few drops” should be changed to “less than one mouthful” to be consistent with other response options on the questionnaire. The altered questionnaire should be validated to ensure ingestion volumes are accurately reported.

## Figures and Tables

**Table 1:** Age and gender distributions of study participants.

	Participant Demographics *n* = 38 (%)
**Age**	
≤18 years	17 (44.7)
>18 years	21 (55.2)
**Gender**	
Male	25 (65.7)
Female	13 (34.2)

**Table 2:** Number of swimmers reporting and actually ingesting pool water amounts within each volume range listed on the questionnaire (*n* = 38).

		No Water–Few Drops (0–26 mL)	One to Two Mouthfuls (27–54 mL)	Three to Five Mouthfuls (55–135 mL)	Six to Eight Mouthfuls (136–216 mL)
Measured Ingestion *	No water–few drops	11 ^†^	14	6	0
	One to two mouthfuls	0	4 ^†^	0	0
	Three to five mouthfuls	0	2	1 ^†^	0
	Six to eight mouthfuls	0	0	0	0

* Measured ingestion values have been categorized using mouthful volumes characterized by Schets et al. [17]. ^†^ Study participants correctly reporting ingestion volume.

## Supplementary Materials

The following are available online at http://www.mdpi.com/1660-4601/15/11/2435/s1.
