# Supplementary material for: Validation of Questionnaire Methods to Quantify Recreational Water Ingestion
Source: Int J Environ Res Public Health. 2018 Nov 1;15(11):2435. doi: 10.3390/ijerph15112435 (PMC6265721; doi:10.3390/ijerph15112435)
Supplement: Supplementary file 1 [file ijerph-15-02435-s001.pdf]

Supplementary Materials

Table 1: Question and answer choices presented to participants. Non-multiple choice questions are followed by bracketed response instructions.

**Question and response options**
What is your sex? [Fill in]
What is your age? [Fill in]
List your weight in pounds [Fill in]
List your height in feet and inches [Fill in]
List your race [Fill in]
List your ethnicity [Fill in]
Select the type(s) of pool(s) you swam in for this study [Select all that apply]<br>1. Wading pool<br>2. Spa<br>3. Wave pool<br>4. Flume<br>5. Therapy pool<br>6. Other
Was the pool indoor or outdoor? [Select yes, no]
How much time did you spend swimming at the pool (min)? [Fill in]
Did you submerge your body at the pool? [Select yes, no]
How long did you swim in the pool (min) [Fill in]
Select all activities you engaged in at the pool [Select all that apply]<br>1. Splashing<br>2. Standing<br>3. Swimming in the deep end<br>4. Swim lessons<br>5. Lap swimming (competitive)<br>6. Lap swimming (non-competitive)<br>7. Used a fountain or other pool play feature<br>8. Used a waterslide<br>9. Diving<br>10. Water aerobics<br>11. A water sport other than competitive swimming<br>12. Other
How long did you engage in each activity listed in the previous question (min)? [Fill in]
When you swam in the deep end, how many times did you swim near the pool

bottom? [Fill in]
Did you wear goggles at any point during the swim? [Select yes, no]
Did you get water up your nose? [Select yes, no]
What is your swimming skill level? [Select one]<br>1. Beginner<br>2. Moderate<br>3. Advanced
During the swim, did you use any of the following swim aids? [Select all that apply]<br>1. Lifejacket<br>2. Kickboard<br>3. Arm floats<br>4. Other swim aid
Are you toilet trained? [Select yes, no]
Did you consume any food at the pool? [Select yes, no]
Did you drink from a drinking fountain at the pool? [Select yes, no]
Did you use the restroom at the pool? [Select yes, no]
Did you change a diaper at the pool? [Select yes, no]
If you changed a diaper at the pool, where did you change the diaper? [Fill in]
Did you smell chlorine at the pool? [Select yes, no]
If you smelled chlorine at the pool, how strong was the chlorine smell? [Select one]<br>1. Weak (barely noticeable)<br>2. Noticeable, but not strong<br>3. Strong
Did you shower before entering the pool? [Select yes, no]
If you showered before entering the pool, how long was the shower (min)? [Fill in]
Have you swam at any pool in the past [select all that apply]<br>1. Two weeks<br>2. Four days<br>3. Three days<br>4. Two days<br>5. Day
How much water did you swallowed while swimming (estimate)? [Select one]<br>1. No water or only a few drops<br>2. 1 to 2 mouthfuls (amount in a shot glass)<br>3. 3 to 5 mouthfuls (amount in a coffee cup)<br>4. 6 to 8 mouthfuls (amount in a soda glass)
What was your age when you visited a pool for the first time (years)? [Fill in]
On average, how much time do you spend at any pool per visit (hours)? [Fill in]

On average, how many times in one year do you visit any pool facility? [Fill in]

Are you currently experiencing any of the following respiratory, eye or ear irritation symptoms? [Select all that apply]

1. Coughing
2. Wheezing
3. Chest tightness
4. Shortness of breath
5. Frequent sneezing
6. Itchy, runny nose
7. Sore throat
8. Backache
9. Eye irritation or stinging
10. Watery eyes
11. Halo vision (halos around lights)
12. Blurry of foggy vision
13. Blue-gray vision
14. Ear infection

Other exposures and health conditions [Select all that apply]

1. Smoker (occasional and regular)
2. Ex-smoker
3. Doctor diagnosed asthma
4. Wheezing that limits daily activities
5. Hay fever
6. Non-drug allergies (other than hay fever)
7. Chronic Obstructive Pulmonary Disease (COPD)
8. Chronic bronchitis (cough and phlegm for at least months a year)
9. Cystic fibrosis
10. Sinusitis (a cold that has not improved after ~7 days of coughing, fatigue, fever, headache, sore throat, or nasal congestion)
11. Flu (combination of fever, cough, muscle/body aches, sore throat, fatigue, runny/stuffy nose lasting 24 hours to ~7 days)
12. Diarrhea anytime in the past 2 weeks (14 days)
13. Crohn's Disease
14. Irritable Bowel Syndrome
15. Ulcerative colitis
16. Partial removal of stomach or intestines
17. HIV/AIDS
18. Hepatitis

19. Eczema or atopic dermatitis
If you selected diarrhea as a health condition, has the diarrhea resulted in 3 or more loose stools in the past 24-hours? [Select yes, no]
If you selected diarrhea as a health condition, has the diarrhea impaired your daily activities (remained at home or in bed)? [Select yes, no]
